# Effects of cocktail of four local Malaysian medicinal plants (*Phyllanthus spp.*) against dengue virus 2

**DOI:** 10.1186/1472-6882-13-192

**Published:** 2013-07-26

**Authors:** Sau Har Lee, Yin Quan Tang, Anusyah Rathkrishnan, Seok Mui Wang, Kien Chai Ong, Rishya Manikam, Bobby Joe Payne, Indu Bala Jaganath, Shamala Devi Sekaran

**Affiliations:** 1 Department of Medical Microbiology, Faculty of Medicine, University Malaya, Kuala Lumpur, Malaysia; 2 Institute for Medical Molecular Biotechnology, University of Islamic Technology Malaysia, Kuala Lumpur, Malaysia; 3 Department of Molecular Medicine, Faculty of Medicine, University of Malaya, Kuala Lumpur, Malaysia; 4 Department of Trauma & Emergency Medicine, UMMC, Kuala Lumpur, Malaysia; 5 American College of Veterinary Pathology, Madison, USA; 6 Biotechnology Centre, Malaysia Agricultural Research and Development Institute (MARDI), Serdang, Malaysia

**Keywords:** *Phyllanthus*, Dengue, Antiviral

## Abstract

**Background:**

The absence of commercialized vaccines and antiviral agents against dengue has made the disease a major health concern around the world. With the current dengue virus transmission rate and incidences, the development of antiviral drugs is of vital need. The aim of this project was to evaluate the possibility of developing a local medicinal plant, *Phyllanthus* as an anti-dengue agent.

**Methods:**

Cocktail (aqueous and methanolic) extracts were prepared from four species of *Phyllanthus* (*P.amarus, P.niruri, P.urinaria,* and *P.watsonii)* and their polyphenolic compounds were identified via HPLC and LC-MS/MS analysis. MTS assay was then carried out to determine the maximal non-toxic dose (MNTD) of the extracts, followed by screening of the *in vitro* antiviral activity of aqueous cocktail extracts against DENV2 by means of time-of-addition (pre-, simultaneous and post-) using RT-qPCR. The differentially expressed proteins in the treated and infected cells were analysed with two dimensional gel electrophoresis experiments.

**Results:**

Several active compounds including gallic acid, geraniin, syringin, and corilagen have been identified. The MNTD of both aqueous and methanolic extracts on Vero cells were 250.0 μg/ml and 15.63 μg/ml respectively. *Phyllanthus* showed strongest inhibitory activity against DENV2 with more than 90% of virus reduction in simultaneous treatment. Two-dimensional analysis revealed significantly altered levels of thirteen proteins, which were successfully identified by tandem MS (MS/MS). These altered proteins were involved in several biological processes, including viral entry, viral transcription and translation regulations, cytoskeletal assembly, and cellular metabolisms.

**Conclusions:**

*Phyllanthus* could be potentially developed as an anti-DENV agent.

## Background

Dengue virus (DENV), of the family *Flaviviridae*, is the causative agent for the high morbidity rated disease- dengue. Being a tropical/sub-tropical disease, dengue is currently endemic in more than 100 countries around the world. It is estimated that 36 million dengue fever cases and another 2.1 million cases of severe dengue occur yearly [1]. These numbers are predicted to increase over the years, mainly due to global warming and the increased frequencies of migration, local and international travelling [2] as this arthropod-borne virus is mainly transmitted by mosquitoes, *Aedes aegypti* and *Aedes albopictus.*

DENV is a single-stranded positive sense RNA virus with a genome of approximately 11 kb [3] which encodes for 3 structural proteins (capsid (C), envelope (E) and membrane (M)) and 7 non-structural (NS) proteins (NS1, NS2A, NS2B, NS3, NS4A, NS4B and NS5). Despite extensive research on dengue, many ambiguities lie in the functions on the various DENV proteins. Nevertheless, the NS3 protein has been shown to be multifunctional with protease activity at its N-terminal together with NS2B and RNA helicase, a nucleoside triphosphatase, and RNA 5′-triphosphatase at its C-terminal [3,4], while, the N-terminal of NS5 represents a methyltransferase and its C-terminal a RNA-dependent RNA polymerase. NS1, on the other hand, has been suggested to play a role in endothelium dysfunction in dengue [5].

Traditionally classified as dengue fever (DF), dengue haemorrhagic fever (DHF) and dengue shock syndrome (DSS), at present, the disease classification has been revised [6] into dengue with/without warning signs and severe dengue. The usual symptoms of dengue include fever, nausea, rashes, whereas the warning signs for the disease includes abdominal pain, persistent vomiting, mucosal bleeding, hepatomegaly and increased levels of hematocrit concurrent with the decreased levels of platelets. Patients who suffer from severe plasma leakage, haemorrhage and organ impairment have higher mortality rates, if not managed well clinically. A primary infection of dengue confers life-long protection towards that particular serotype, not towards the other 3 DENV serotypes [2]. This particularly complicates secondary infections, where antibody enhancement [7] and cross-reactive memory T cell activation [8] are postulated to cause more severe manifestations of dengue. To date, there are neither effective anti-dengue agents nor commercially licensed vaccines for the treatment of dengue [9,10]. The current methods of controlling dengue are mosquito eradication programs [11] and fluid management therapy for infected individuals [6]. Therefore, the need for new antiviral agents is imperative for early treatment to prevent manifestation of severe dengue, to curb outbreaks and in the future to complement possible vaccination programs.

Plants have always been an exclusive part in traditional healing, and in the past decades as treatment sources for various diseases, due to their complex bioactive ingredients and rich source of pharmaceuticals [12]. Plants have been revealed to offer better sources of antiviral agents compared to synthetic analogues [13], which has since prompted identification of more than 3800 plants with efficient abilities to suppress the growth of numerous viruses [14]. These include *Olea eurolaea* leaf extract which demonstrated strong antiviral activity towards human immunodeficiency virus (HIV) type 1 [15] and influenza virus [16], *Rozites caperata* which acts against herpes simplex virus (HSV) types 1 and 2 [12], *Sargassum patens* acting against HSV type 1 [17], as well as *Phyllanthus nanus*, *Salvia miltiorrhiza*, *Radix astragala*, and *Rheum palmatum* which have anti-hepatitis B virus effect [18]. The most intriguing fact of using plant sources as antiviral agents, lies in the fact that these plants are usually found in abundance in developing and third world countries, where infectious diseases occur are more rampant. One such plant genus is the *Phyllanthus* (Euphorbiaceae), which has around 750 species [19] and is distributed throughout the tropical and subtropical countries (which are also where dengue occurs). *Phyllanthus* is well known for its pharmacological properties in folk medicine [20] as well as in modern treatments of kidney and liver diseases, urinary bladder and intestinal infections; diabetes, hepatitis, dysentery, jaundice, gonorrhea and skin ulcers [20,21]. The plant has also been proposed to show anti-cancer activities [22,23].

With tremendous potential as pharmacological sources, we seek to evaluate the antiviral potential of *Phyllanthus* cocktail extracts which is a mixture of four *Phyllanthus* species, namely *P. urinaria, P. niruri, P. amarus,* and *P. watsonii in vitro* against dengue virus type 2 (DENV2) as well as to determine its possible pharmacological activity.

## Methods

### Cocktail extract preparation

The cocktail (aqueous and methanolic) extracts consisting of four different pooled *Phyllanthus* species (*P.amarus, P.niruri, P.urinaria, and P.watsonii*) were kindly prepared and provided by the Biotechnology Centre, Malaysian Agricultural & Research Development Institute (MARDI). All four species of *Phyllanthus* were grown in greenhouse from year January 2008 till December 2010 under controlled irrigation and fertilization at MARDI and the samples were harvested every two months since March 2008. The plant species were identified based on taxonomical identification by Dr Salma Idris, a taxonomist from Strategic Research Centre, MARDI and the herbarium specimen is kept at MARDI, Serdang. Each of the species contains different chemical profiles in terms of the type of phytochemicals and their amount. Hence, a cocktail *Phyllanthus* extract was prepared from *P.watsonii*, *P. amarus*, *P. niruri*, and *P. urinaria* in the ratio of 2:2:1:1 respectively, as we wanted to obtain a wide range of phytochemicals that will eventually hit multiple disease targets. We hope that the synergistic effects of the various phytochemical from the four species will be able to be more effective in challenging the disease through its multiple targets.

Briefly, whole plant samples (minus roots) were freshly harvested, washed and dried at room temperature. The samples were cut into smaller pieces and ground with liquid nitrogen into powder and finally freeze dried. For aqueous extract, freeze-dried powder was extracted with 20 mL of water, 20 mM of diethyldithiocarbamic acid and 0.5% formic acid, whereas, absolute methanol was used for methanolic extract preparation. The samples were subjected to homogenization and filtered with 15 Whatman No. 40. The extracts were stored at −20°C until use and a single batch of extracts was used for all the experiments.

### Identification of proteins through high performance liquid chromatography (HPLC) and mass spectrometry (LC-MS-MS) analysis

Proteins were identified using HPLC and LC-MS-MS as previously described by Tang and colleagues [22]. For the aqueous cocktail extract, 2 ml of supernatant was concentrated by vacuum evaporator, followed by re-dissolving in 20 mg/ml with 30% methanol and then subjected to LC-MS-MS. While for the methanolic extract, supernatant was subjected to rotary evaporation (Rotavapor RII, BUCHI, Switzerland) and re-dissolved with 20% methanol. The sample was then separated using solid phase extraction (SPE) column (LiChrolut RP-18 1000 mg/6 ml, Merck Germany) with the mobile phase of 60% and 70% methanol. The collected elutes were then concentrated to a volume of 0.5 ml, then diluted eight times with 40% methanol, and finally subjected to LC-MS-MS analysis.

The separation of both extracts was carried out using HPLC binary pump, an autosampler injector compartment and a diode array detector (DAD) (1200 Series, Agilent Technologies, Germany). The reverse phase used was C-18, 150 mm X 4.6 mm i.d. 5 μm particle size of Thermo Hypersil GOLD column (Thermo Scientific, UK), while the composition of mobile phase used was 0.1% formic acid in water (solvent A) and 0.1% formic acid in acetonitrile (solvent B) with gradient for solvent B: 5% (5 min), 5-90% (60 min), 5% (4 min) at a flow rate of 1 ml/min. Total volume of injection was 20 μl and the detection was assigned at 280 nm and 360 nm. For mass spectrometry analysis, 3200 QTrap LC/MS/MS system (Applied Bioscience – MDS Sciex) was utilized. For negative ionization, the iron source was set at 500°C and voltage maintained at −4.5 kV. The nitrogen generator was operated at 60 curtain gas flow, 90 psi source gas flow and 60 psi exhaust gas flow. Two scanning modes were used; enhance mass spectrometer (EMS) and enhance ion product (EPI) for a full scan mass spectra ranging *m/z* 100–1200.

### Virus and cell culture

The New Guinea C (GeneBank Accession No. M29095) strain of DENV2 was obtained from the Department of Medical Microbiology, University of Malaya and used throughout the study. The virus was cultured and propagated in *Aedes albopictus* mosquito cell line C6/36 (ATCC: CRL-1660) with Leibowitz-15 (L-15) medium containing 1% heat-inactivated foetal bovine serum (FBS. Gibco) at 30°C. The supernatant from infected C6/36 cells was collected for viral titre determination as well as for subsequent experimental applications. The porcine kidney (PS) cell line was maintained in L-15 media containing 5% FBS at 30°C and the African green monkey kidney cells (Vero) (ATCC: CCR-81) were cultured in DMEM supplemented with 10% FBS at 37°C. Cells were maintained in humidified air with 5% CO_2_. Cells undergoing exponential growth were used throughout the experiments.

### Virus titration

The PS cells were seeded at a cell density of 1.5 × 10^5^ per well in 24-well plates (Nunc, Denmark). Ten-fold serial dilutions of viral supernatant were prepared in L-15 medium containing 1% FBS, and loaded on the cells. After 3 hours, 3% carboxylmethyl cellulose (Calbiochem, USA) containing 3% FBS in L-15 was added into each well and further incubated for 3–4 days. After 3–4 days of incubation, the cells were washed with PBS, and stained with 1% of naphthalene blue, followed by visual counting of plaques.

### Determination of maximum non-toxic dose (MNTD)

Prior to screening of *Phyllanthus* cocktail extracts for their antiviral properties, they were first subjected to cytotoxicity assay to identify the maximal non-toxic dose (MNTD) on Vero cells. The MNTD is a concentration where it does not show any toxicity effects on cell viability. Briefly, 1 × 10^4^ Vero cells were seeded into each well of a 96-well flat-bottom plate and incubated overnight for attachment. After 24 hours, different concentrations of extracts ranging from 15.63-500 μg/ml were added into respective wells in triplicates and further incubated for different time points (24, 48 and 72 hours). Control wells contained cells with culture medium without the extracts and negative control wells contained only culture medium with different concentrations of the extracts. After each time course of study, 100 μl of supernatant was discarded and 20 μl of MTS/PMS solution (Promega, USA) was added, followed by incubation in the dark for 1 hour. Absorbance was measured using GloMax (Promega, USA) at 490 nm with a reference wavelength of 600 nm. Absorbance is directly proportional to the number of live cells in the culture. Thus, the dose–response graph was presented in percentage of Vero cell’s viability against different concentration of extracts.

### *In vitro* antiviral experiment

Three different modes of treatment were performed to study the *in vitro* antiviral activity of *Phyllanthus*. For pre-treatment mode, Vero cells were seeded in a 24-well plate and incubated overnight for attachment. Before virus inoculation, the MNTD of *Phyllanthus* extract was added to the cells and incubated for 24 h. DENV2 was inoculated at an MOI of 0.1 onto the *Phyllanthus*-treated Vero cells. For the simultaneous treatment, *Phyllanthus* extract and DENV2 (MOI: 0.1) were prepared and then inoculated onto near confluent Vero cell monolayers. In the post treatment mode, DENV2 (MOI: 0.1) were inoculated onto the cells and incubated for 24 h. After 24 hour, the medium was removed and replaced by the *Phyllanthus* extract. Subsequently for all treatment modes, the cultures were further incubated for 24, 48 and 72 h at 37°C under 5% CO_2_ atmosphere. After each time point of study, supernatants as well as cells were collected separately subsequent to 2 cycles of freeze-thawing and were then stored at −80°C for ensuing experiments.

### RNA isolation and real-time RT-PCR

Real time RT-PCR was performed to detect and quantitate viral RNA in infected Vero cells treated with *Phyllanthus* extract. RNA was extracted from samples (supernatant and cells) according to the instructions provided by AccuPrep viral RNA extraction Kit (Bioneer, Korea) and the extracted RNA was then stored at −80°C until further use. Dengue virus standard curve was prepared using MAXIscript Transcription Kit (Ambion, USA). Ten-fold serially diluted RNA was prepared and subjected to real-time RT-PCR. The copy number of RNA was calculated based on the concentration measured and its molecular weight. RT-PCR was carried out using iScript^TM^ One Step RT-PCR Kit with SYBR Green (Bio-Rad, USA), employing dengue group specific primers with adaption of SYBR green technology [24]. The 25 μl reaction mixture containing 5 μl of sample RNA, 0.25 μl of each primers, 0.25 μl of reverse transcriptase enzyme, 12.5 μl of SYBR Green mix and water, was amplified in a CFX96 RT-PCR machine (Bio-Rad, USA). The thermal profile for the one-step SYBR Green-based RT-PCRs consisted of a 1 hour RT step at 45°C, followed by 35 cycles of 3 steps amplification at 95°C for 30 seconds, 60°C for 30 seconds and 72 for 30 seconds. Fluorescence data were collected during the extension step. Negative and positive controls were included in each analytical run.

### Two-dimensional (2D) gel electrophoresis

Equal amounts of total protein from (i) cells only, (ii) *Phyllanthus*-treated cells, (iii) DENV2- infected cells, and (iv) *Phyllanthus* treated-infected cells, were subjected to 2D gel electrophoresis according to the manufacturer’s instructions (GE Healthcare). Briefly, total proteins were extracted from control and experimental samples by incubation with lysis buffer on ice for 30 minutes. The protein pellets were re-solubilized in rehydration solution (8 M urea, 2% CHAPS, 40 mM DTT, 0.5% IPG buffer pH3-11NL, bromophenol blue) and kept at −80°C until further analysis. Total amount of proteins was determined using 2D Quant kit (GE Healthcare) and 250 μg of proteins were rehydrated into 13 cm immobilized pH gradient (IPG) strips (pH 3–11 nonlinear) (GE Healthcare). The first dimension was electrophoresed on the IPGphor III machine (GE Healthcare) at 20°C with the following settings: step 1 at 500 V for 1 hour; step 2 at 500-1000 V for 1 hour; step 3 at 1000-8000 V for 2.5 hour, and step 4 at 8000 V for 0.5 h. Once first dimensional separation was completed, the gel was equilibrated as follows; first reduction with 64.8 mM of dithiothreitol-SDS equilibration buffer (50 mM Tris–HCl [pH 8.8], 6 M urea, 30% glycerol, 2% SDS, 0.002% bromophenol blue) for 15 minutes, followed by alkylation with 135.2 mM of iodoacetamide-SDS equilibration buffer for another 15 minutes. The second dimension electrophoresis was carried out using the SE600 Ruby system (GE Healthcare) at 25°C in an electrode buffer (25 mM Tris, 192 mM glycine, and 0.1% [wt/vol] SDS) with the following settings: step 1 at 100 V/gel for 45 minutes; step 2 at 300 V/gel until the run is completed. The gels used in the second dimension were 12.5% homogenous acrylamide gels casted in the laboratory. After electrophoresis, the gels were fixed with destaining solution for 30 minutes, followed by staining with hot Coomasie blue for 10 minutes. The gels were scanned using Ettan DIGE Imager (GE Healthcare). Gel images were analyzed using PDQuest 2-D Analysis Software (Bio-Rad, USA) and only protein spots which showed significant differences (more than 1.5 fold) were selected for mass spectrometry analysis.

### Protein digestion and desalting and MALDI-TOF/TOF analysis

Protein spots excised from polyacrylamide gels were kept in sterile 1.5 ml tubes and sent to Proteomics International Pty Ltd, Australia for further analysis. Briefly, the protein samples were first digested with mass spectrometry grade typsin and the peptides were extracted according to standard techniques . Peptides were analyzed by MALDI-TOF/TOF mass spectrometer using a 5800 Proteomics Analyzer [AB Sciex]. Identification of proteins was performed by using Mascot sequence matching software [Matrix Science] with Uniprot database.

### Data analysis

Results were expressed as the mean ± standard error mean (SEM) of data obtained from triplicate experiments. All data were analyzed using one way ANOVA, followed by Dunnett’s test for pairwise comparison. P < 0.05 was considered statistically significant for all tests. All analysis was performed using SPSS software.

## Results

### Identification of polyphenol compounds in *Phyllanthus*

**Figure 1:**
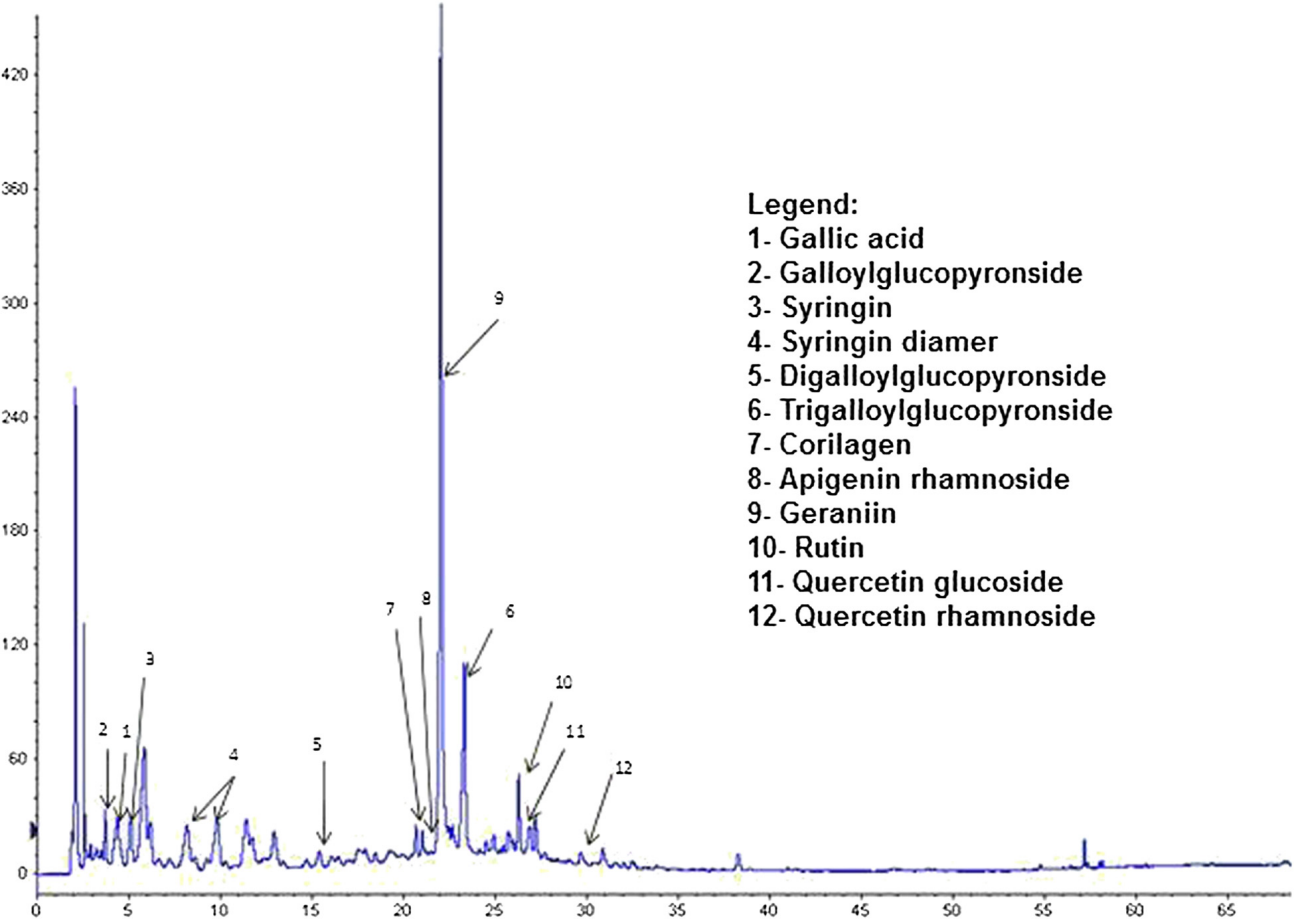
**Separation of polyphenolic compounds from *Phyllanthus *cocktail by reverse-phase HPLC.**

The phytochemicals contained in all *Phyllanthus* species were first optimally separated using HPLC and then identified through LC-MS-MS. To obtain the best cocktail extract, different *Phyllanthus* species were mixed at specific proportions to obtain the highest amounts of health benefiting phytochemicals. The mixed cocktail extracts were analysed using both HPLC and LC-MS-MS where a variety of polyphenol compounds were identified (Figure 1). These compounds include gallic acid, galloylglucopyronside, corilagen, geraniin, rutin, quercetin glucoside, syringing, syringing diamer, digalloylglucopyronside, trigalloylglucopyronside, apigenin rhamnoside, and quercetin rhamnoside. Their individual percentages in both the aqueous and methanolic extracts are shown in Table 1 and among this compounds, geraniin constitutes the greatest amount in both the extracts, in particular in the methanolic extract.

**Table 1:** Percentages of individual polyphenolic compound in aqueous and methanolic extracts

**Location of compounds found in chromatogram**	**Compound**	**% present in water extract**	**% present in methanolic extract**
1	Gallic acid	0.0004	0.032
2	Galloylglucopyronside	0.0003	Not detected
3	Syringin	0.0005	Not detected
4	Syringin diamer	0.0005	Not detected
5	Digalloylglucopyronside	0.0002	0.019
6	Trigalloylglucopyronside	0.0003	0.020
7	Corilagen	0.0008	0.084
8	Apigenin rhamnoside	<0.0002	Not detected
9	Geraniin	3.237	17.140
10	Rutin	0.015	0.012
11	Quercetin glucoside	0.021	0.016
12	Quercetin rhamnoside	<0.0002	Not detected

### Cytotoxicity activity of aqueous and methanolic extracts of *Phyllanthus*

**Figure 2:**
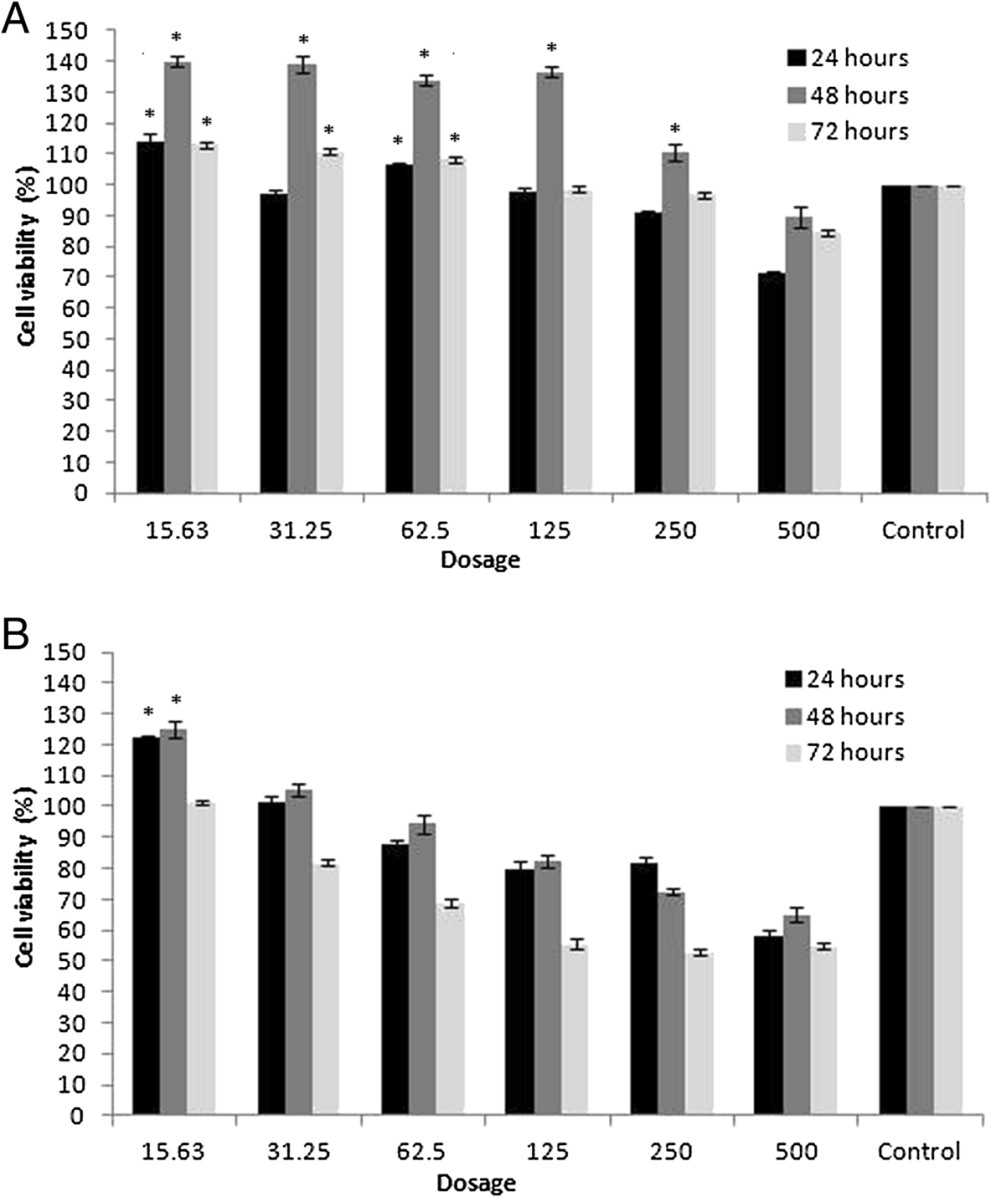
**Percentage of Vero cells viability at different times of incubation and dosages of *Phyllanthus *treatments. (A)** Vero cells treated with aqueous cocktail extract of *Phyllanthus* and **(B)** Vero cells treated with methanolic cocktail extract of *Phyllanthus*. Results are expressed as mean ± SEM of three independent experiments. * signifies *p* < 0.05 compared to the untreated control.

From the cytotoxicity study using the Vero cell line, we noted that the MNTD for aqueous and methanolic extracts were 250 μg/ml and 15.63 μg/ml, respectively (Figures 2A and 2B). The aqueous cocktail extract of *Phyllanthus* showed lower toxicity to Vero cells as compared to the methanolic extract as its cell viability remained above 90% when treated up to 250 μg/ml. Thus, only the aqueous cocktail extract of *Phyllanthus* was selected for further antiviral studies.

### *In vitro* antiviral activity of *Phyllanthus*

**Figure 3:**
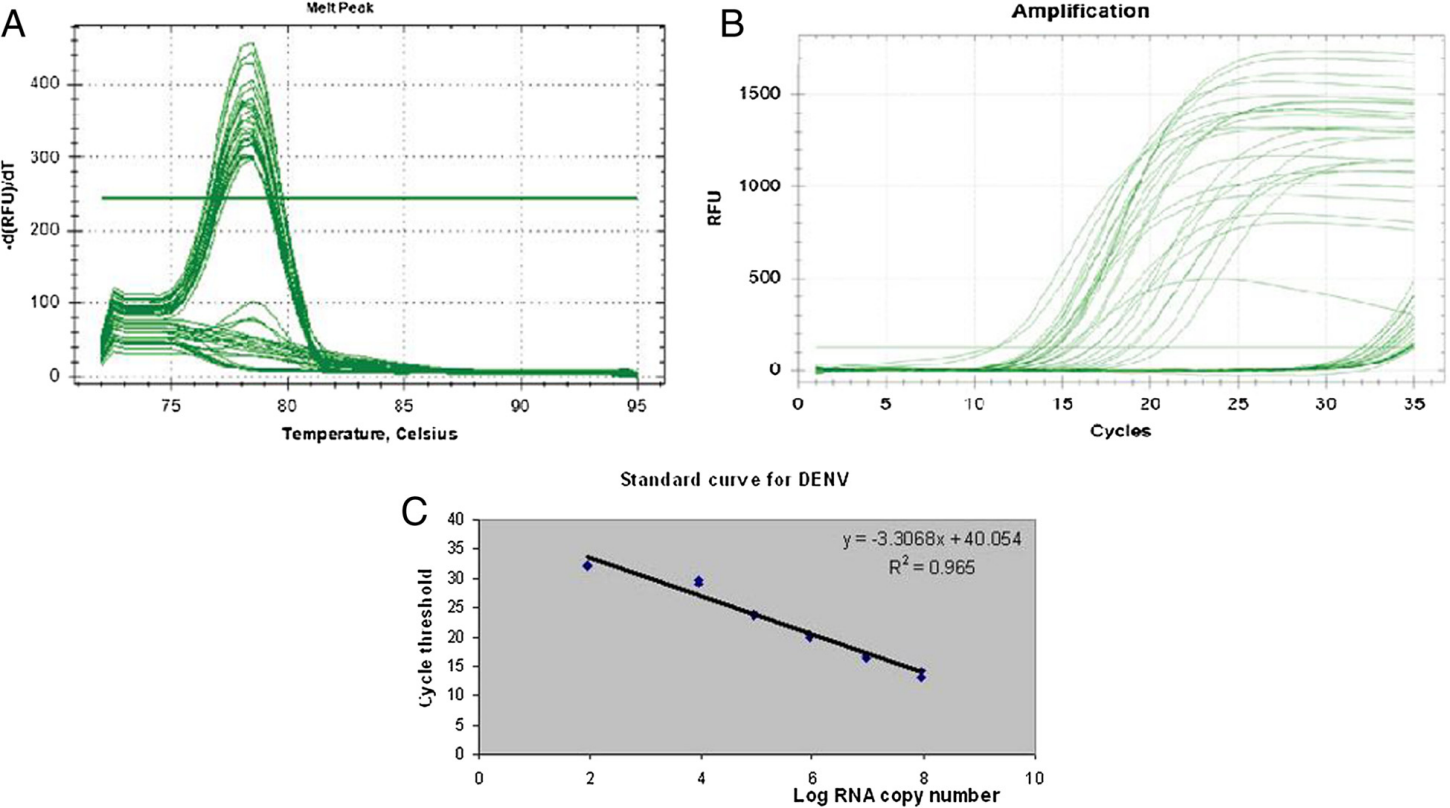
**In-vitro antiviral assay via SYBR-Green RT-PCR. (A)** Melt peak of DENV2. **(B)** Representative amplification plot for DENV2 antiviral assay. **(C)** Standard curve for DENV2.

Via real time RT-qPCR, the *in vitro* antiviral activities of aqueous *Phyllanthus* cocktail on DENV2 were assessed in 3 different treatment modes (Figure 3, Additional file 1). Comparing the three different modes of treatment, *Phyllanthus* showed the strongest antiviral activity against DENV2 during the simultaneous mode of treatment with more than 83 - 95% reduction of virus inhibition (Table 2). The morphological alterations of Vero cells due to virus replication were completely prevented when 250 μg/ml of the extract was added into the infected cultures. On the other hand, the virus reduction was not pronounced in pre-treatment as compared to simultaneous treatment, with only 5 - 46% virus inhibition in both supernatant and cell samples. Contrarily, no virus reduction with post-infection treatment with *Phyllanthus* extracts.

**Table 2:** Percent Inhibition of virus copy number in cell and supernatant at three different treatment modes

**Percent Inhibition of virus copy number (%)**						
**24 Hour**			**48 Hour**		**72 Hour**	
**Cell**		**Supernatant**	**Cell**	**Supernatant**	**Cell**	**Supernatant**
**PRE-TREATMENT**						
**C + V**	0	0	0	0	0	0
**C + P + V**	6.38 ± 5.59	46.07 ± 0.88^*^	4.66 ± 6.33	39.01 ± 5.01^*^	0	17.87 ± 4.89^*^
**SIMULTANEOUS TREATMENT**						
**C + V**	0	0	0	0	0	0
**C + P + V**	94.69 ± 0.40^*^	82.85 ± 3.91^*^	92.78 ± 0.83^*^	84.61 ± 6.58^*^	93.61 ± 1.38^*^	91.48 ± 3.66^*^
**POST-TREATMENT**						
**C + V**	0	0	0	0	0	0
**C + P + V**	0	0	0	0	0	0

Results are expressed as mean ± SEM of three independent experiments. C + V = dengue-infected cells; and C + P + V = *Phyllanthus* treated-dengue infected cells. * signifies *p* < 0.05.

### Proteome analysis of DENV2 infected cells with or without *Phyllanthus* treatment

**Figure 4:**
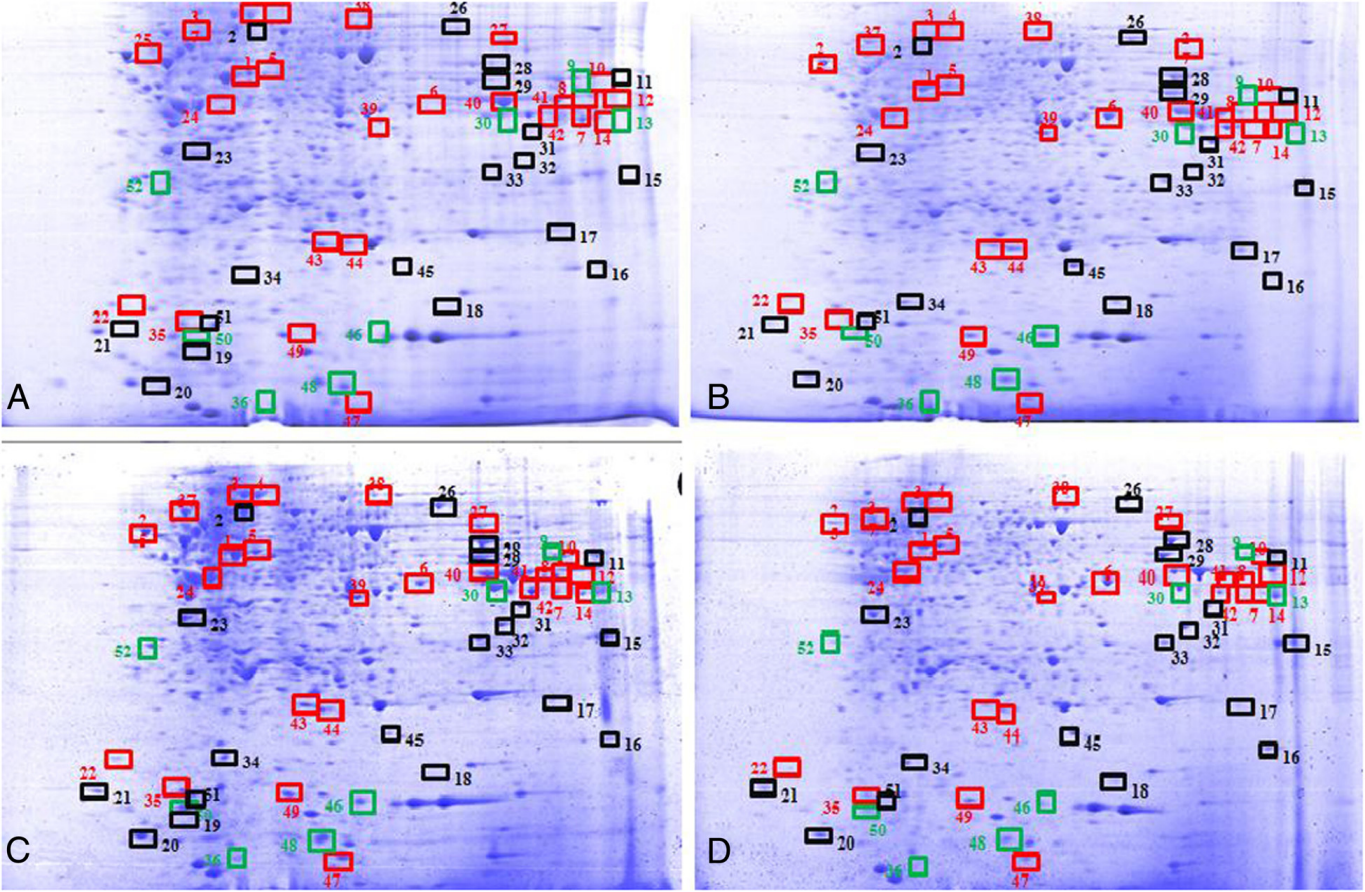
**Proteome analysis of DENV2 infected cells with or without *Phyllanthus *treatment. (A)** Cells Only, **(B)***Phyllanthus*-treated cells, **(C)** DENV2 Infected cells and **(D)***Phyllanthus* treated infected cells.

**Figure 5:**
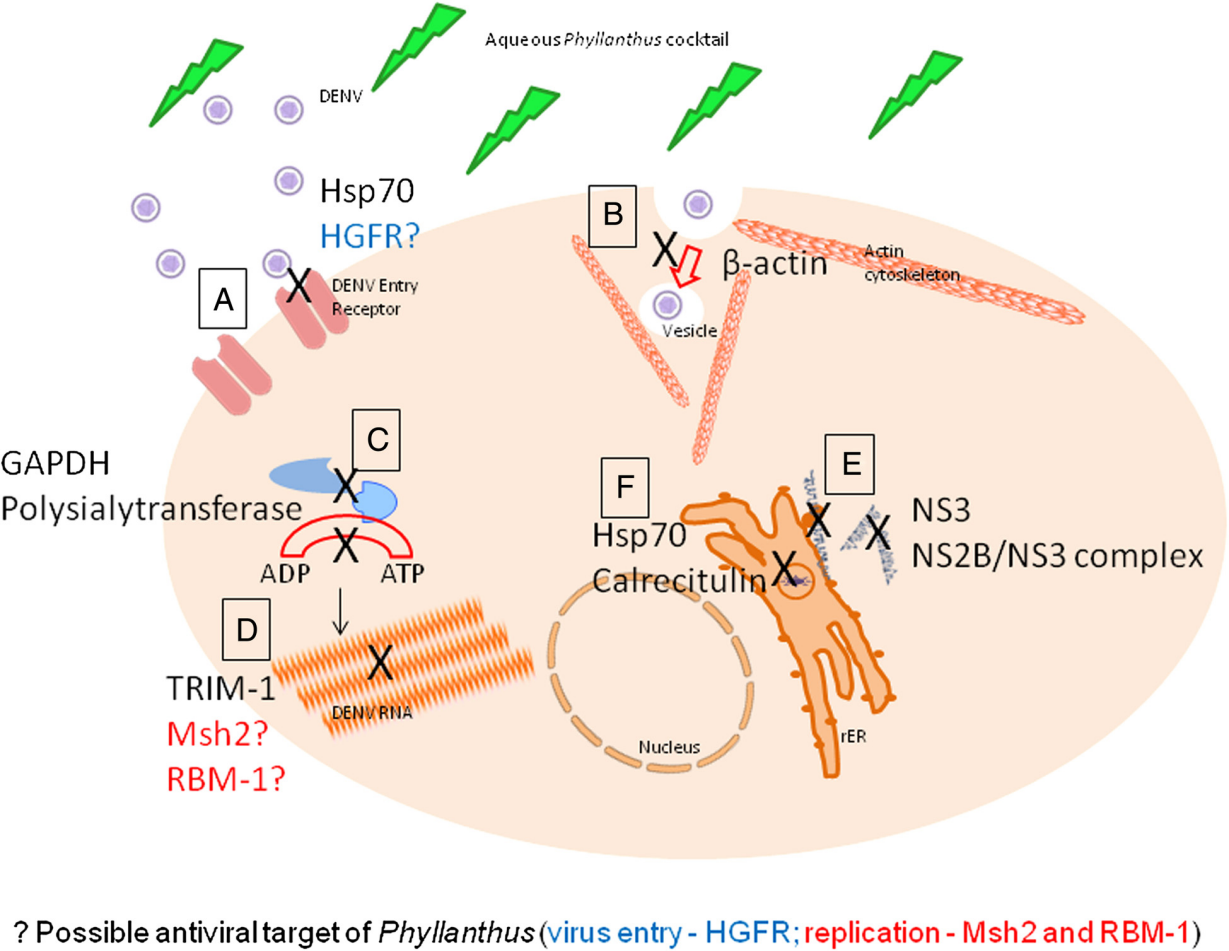
**Schematic diagram showing possible mechanism of dengue virus inhibition by *Phyllanthus*. (A)** Viral entry; **(B)** Viral endocytosis, membrane fusion, necleocapsid release; **(C)** Glucose uptake; **(D)** Viral pre replication and RNA replication; **(E)** Viral production and proteolytic processing; and **(F)** Viral assembly and maturation.

To determine the actual antiviral mechanisms of *Phyllanthus* against DENV2 protein profiling of *Phyllanthus*-treated dengue-infected cells via 2D gel electrophoresis was carried out. This was compared to three other different protein profiles including cells only, *Phyllanthus*-treated cells and dengue-infected cells. A total of 52 proteins were found to be differentially expressed (Figure 4), but only 24 proteins were deemed significant (protein scores > 55) after mass spectrometry analysis. Fourteen proteins which had significantly altered expression were identified, where some of these proteins are represented by more than one protein spot (Table 3). These include Calreticulin, Trim1, Heat shock 70 kDA protein,, Beta actin, Hepatocyte growth factor receptor, DNA topoisomerase I, NS3, G3PD (Glyceraldehyde-3-phosphate dehydrogenase), RBM1 (RNA-binding motif 1), Histidine triad nucleotide-binding protein 1, DNA mismatch repair protein Msh2, Chain B, Dengue virus NS2bNS3, and Polysialyltransferase. These proteins consist of host cell proteins as well as viral proteins that are involved in various biological processes, including viral entry and replication, molecular chaperoning, cytoskeletal assembly, and cellular metabolisms (Table 3; Figure 5).

**Table 3:** **Differential protein levels in*Phyllanthus*-treated dengue-infected Vero cells**

**ID**	**Accession**	**Score**	**Possible proteins**	**C**	**C + P**	**C + V**	**C + V + P**
**Viral Entry**							
3	Q28222.1	1059	Heat Shock Protein 70 (Hsp70)	192.1	200.2	312.2	234.3
4	Q28222.1	768	Heat Shock Protein 70 (Hsp 70)	199.5	287.4	419.3	282.2
5	Q2IBA6.1	483	Hepatocyte Growth Factor Receptor (HGFR)	192.7	200.4	313.4	259.7
12	Q2IBA6.1	136	Hepatocyte Growth Factor Receptor (HGFR)	222.5	0.0	313.2	218.2
14	Q2IBA6.1	178	Hepatocyte Growth Factor Receptor (HGFR)	206.3	0.0	312.5	200.1
22	Q2IBA6.1	139	Hepatocyte Growth Factor Receptor (HGFR)	231.9	237.1	331.3	225.5
39	Q2IBA6.1	132	Hepatocyte Growth Factor Receptor (HGFR)	203.8	206.7	390.0	212.9
41	Q2IBA6.1	120	Hepatocyte Growth Factor Receptor (HGFR)	211.0	224.2	333.2	221.6
**Viral Uncoating**							
37	AAT48107.1	132	Trim 1	187.4	198.8	366.1	535.3
**Viral Transcription and Translation**							
7	ABB92440.1	489	RNA Binding Motif 1 (RBMI)	197.6	0.0	412.3	222.2
8	ABB92440.1	383	RNA Binding Motif 1 (RBMI)	206.0	0.0	362.1	226.7
10	ABB92440.1	141	RNA Binding Motif 1 (RBMI)	213.8	0.0	372.7	221.2
24	ABB92440.1	77	RNA Binding Motif 1 (RBMI)	180.4	0.0	362.4	181.7
42	ABB92440.1	633	RNA Binding Motif 1 (RBMI)	202.9	222.2	424.2	313.3
27	NP_739587.2	173	Nonstructural protein NS3	0.0	0.0	312.3	123.2
35	2FOM_B	151	Chain B, Dengue virus NS2bNS3 Protease	0.0	0.0	262.8	123.2
38	Q7YR26.1	63	DNA topoisomerase 1 (DNA Topo 1)	202.9	211.3	373.2	229.2
43	Q5XXB5.1	84	DNA mismatch repair protein Msh2	201.1	212.2	415.2	311.3
44	Q5XXB5.1	129	DNA mismatch repair protein Msh2	209.1	205.3	394.2	213.3
47	Q5RF69	185	Histidine triad nucleotide-binding protein 1	194.4	189.1	393.4	194.0
**Viral Post-translational Modification**							
25	Q4VIT5.1	346	Calreticulin	188.0	200.4	333.3	213.2
**Glucose Uptake and Glycolytic Enzymes**							
6	Q8CGV6	559	Glyceraldehyde-3-phosphate dehydrogenase (G3PD)	213.9	231.3	524.1	313.2
49	AAF17105.1	151	Polysialyltransferase	207.1	212.3	313.7	232.3
**Cytoskeletal assembly**							
1	Q76N69.1	846	Beta actin	168.8	185.3	512.2	273.2

C = cells only; C + P = *Phyllanthus*-treated cells; C + V = dengue-infected cells; C + P + V = *Phyllanthus* treated dengue infected cells.

## Discussion

Since the discovery of *Phyllanthus* and its anti-hepatitis B viral effect in the late 1980s [25], the plant *Phyllanthus* has been tested for its various pharmacological effects. Various species of this plant genus have exhibited activities that range from being anti-hepatotoxic [26,27], antiviral [25,28], antibacterial [29,30], anti-diabetic [31,32] to having anti-tumour and anti-carcinogenic effects [22,23]. Here, we assess the effectiveness of *Phyllanthus* against DENV which has become a global menace especially in the lack of commercialized vaccines and anti-DENV agents.

Two different *Phyllanthus* cocktail of different extraction method was tested initially for their toxicity against Vero cells *in vitro*. The aqueous cocktail displayed lower cytotoxicity even up to the concentration of 250 μg/ml and was thus selected for subsequent experimental procedures. The *Phyllanthus* cocktail had various bioactive compounds which included gallic acid, geraniin, syringing and corilage (Figure 1) [22,23] and these components are believed to behave synergistically to display the anti-DENV activity observed in this study. Nevertheless, the percentages of geraniin in the extracts were the highest and hence were predicted to exert most of the antiviral effect. Yang et al. (2007) had demonstrated the suppression of herpes simplex viruses 1 and 2 by geraniin isolated from *Phyllanthus urinaria*[33]. Besides that, geraniin was also demonstrated to inhibit enterovirus 71 replication in both *in vitro* and *in vivo*[34]. These studies therefore accentuate the potential of *Phyllanthus* cocktail to act as an agent that suppresses dengue virus infection due to its presence of high geraniin content.

The DENV replication cycle involves several steps: (1) viral attachment; (2) viral entry; (3) membrane fusion; (4) RNA release; (5) viral protein production; (6) RNA replication; (7) viral assembly; (8) viral transport and maturation and lastly (9) viral release. Hence, the anti-DENV assessment of the aqueous *Phyllanthus* was done in a time-of-addition manner and we noted that the extract exhibited the most activity in the simultaneous assay, which was then followed by the pre-treatment assay. This implied that the *Phyllanthus* extract may be blocking viral adsorption into the Vero cells [35]. On the other hand, post-treatment of Vero cells following DENV infection showed minimal antiviral effects, indicating that it possibly had lower effect on the late replication stage of DENV RNA replication. Therefore, we postulated that firstly *Phyllanthus* could possibly be altering/blocking the virus or host factors [36] by hindering viral entry into the host cells. Secondly, this inhibition could also be partly due to the ability of some bioactive components in the extract which can directly inactivate DENV particles [37].

In order to obtain a clearer picture behind the anti-DENV of *Phyllanthus* aqueous cocktail, we performed a protein profiling assay to study protein alterations in *Phyllanthus* treated DENV infected Vero cells. Of the many differentially expressed proteins observed, 13 different proteins were significantly noted to play roles in DENV reduction. As postulated, several of the proteins differentially expressed were noted to play roles in viral adsorption and entry. Host cell receptors for DENV include heparan sulphate; Grp78 on HepG2 cells and DC-SIGN on macrophages and monocytes. The monocytic cells also consist of Hsp70 and Hsp90 which are important components of the lipid raft in the DENV entry receptor complex [38,39]. The expression of Hsp70 was decreased in the *Phyllanthus* treated- DENV infected cells, indicating that viral attachment and entry via this particular receptor complex has been thwarted (Figure 5A). The Hsp70 has also been known to interact with LOX1 receptor to initiate cytotoxic T lymphocytes (CTL) response [40]. A reduction of Hsp70 by *Phyllanthus* could prevent activation of cross-reactive memory T cells in a secondary infection, a hypothesized mechanism behind dengue immunopathogenesis.

The hepatocytes are target cells in DENV2 infections [41] and the hepatocyte growth factor (HGF) is a mitogen for hepatocytes [42]. The HGF receptor (HGFR) has been implied as a co-receptor for adeno-associated virus type 2 infections [43], and although this has not been shown for dengue virus entry, the HGF per se has been found to be increased in the serum of dengue infected patients [44]. A down-regulation of the HGFR was noted in the *Phyllanthus* treated DENV2 infected cells indicating a possible role for this receptor for virus entry into cells (Figure 5A).

The cytoskeleton is an important part of a cell and one of its main components is the β-actin which was found to be down-regulated by *Phyllanthus* in DENV2 infected cells (Figure 5B). Dengue viruses enter cells via receptor-mediated endocytosis, form vesicles, undergo membrane fusion and their necleocapsid released into the cytoplasm. These movements are mediated by the actin skeleton [45] and in a DENV2 infection study, the actin skeleton has been shown to be an integral part for viral entry, production as well as release. Therefore, here we show that since *Phyllanthus* has shown inhibitory effects before and during DENV2 infection, it is clear that the plant may be inhibiting viral entry, viral motility in cells or the synthesis of viral polyproteins.

During a viral infection, glucose uptake and glycolytic enzyme activity is usually increased for ATP production [46], a major energy source for cells. The ATPs are necessary in a variety of ATP-dependent cellular processes during viral replication [47] and they are usually catalyzed by viral-encoded enzymes or complexes consisting of viral and host-cell proteins. Among the many enzymes involved, two (glyceraldehyde-3-phosphate dehydrogenase (GAPDH) and polysialyltransferase) were found to be down-regulated in *Phyllanthus* treated DENV2 infected cells (Figure 5C). The GAPDH has also been shown to co-localize with viral RNA-dependent RNA polymerase (NS5) in JEV-infected cells [48] and this may be reflected in DENV infection. The reduction of GAPDH may therefore lead to the inhibition of replication and amplification of viral RNA. Meanwhile, polysialyltransferase is an enzyme that polymerizes the sugar into a polysaccharide form and a reduction of these overexpressed enzymes in *Phyllanthus*-treated infected cells may lead to a low production of ATP and hence, a reduction in viral RNA replication. Generally, the decrease of both these proteins imply that *Phyllanthus* maybe preventing the virus from hijacking the host cell’s glucose uptake and glycolytic enzymes (GAPDH and polysialyltransferase enzyme) during viral replication.

Tripartite motif (TRIM) proteins are involved in diverse cellular processes, such as cell proliferation, differentiation, oncogenesis, and apoptosis [49]. The exact functions of TRIMS during virus infection are still poorly understood but TRIM1 has been shown to display anti-viral activity against N-tropic murine leukaemia virus [50] by inhibiting the pre-reverse transcription step, suggesting a role for this particular protein against viral genome replication. Hence, it is possible that the high levels of TRIM1 protein detected in our *Phyllanthus* treated cells may have halted the genome replication of DENV2 and thus disrupted further viral propagation (Figure 5D).

Proteolytic processing of DENV polyprotein is an important step in viral replication and maturation. The DENV NS3 is multifunctional with a serine protease at its N-terminal, together with NS2B (NS2B/NS3 complex) cleaves the viral polyprotein for the subsequent viral assembly and maturation [51]. The dengue NS3 was detected at lower levels in *Phyllanthus* treated infected cells, and this directly reduced the amount of NS2B/NS3 pro complex formed as was observed in this study (Figure 5E). This complex has been a major and promising target for the development of anti-DENV agents, and *Phyllanthus* has shown this potential by inhibiting polyprotein processing.

Following polyprotein processing, viral budding occurs in the endoplasmic reticulum (ER), where they form immature virion and are then transported to the Golgi complex. The human immunoglobulin binding heavy chain protein, a HSP70 member and 2 ER chaperones, calnexin and calreticulin have been found to be involved in DENV E protein folding and assembly [52]. A reduction of both Hsp70 and calreticulin in our study after treatment with *Phyllanthus*, indicates that this plant is able to disrupt production of mature virions (Figure 5F).

Four other proteins were also detected to be down-regulated by *Phyllanthus* in the DENV2 treated Vero cells, including DNA topoisomerase I, RNA-binding motif (RBM)-1, histidine triad nucleotide-binding protein 1, and DNA mismatch repair protein (Msh2). These proteins’ functions have mainly revolved around the DNA replication of host cells, and are still elusive in the infection of DENV. Nevertheless, the Msh2 DNA mismatch protein have been shown to be required for the replication of HSV-1 and Epstein-Barr virus, where the protein has been localized to the viral replication compartments [53,54]. The RBM-1 is also known as heterogeneous ribonuclearprotein (hnRNP)- G. Several protein of the hnRNP family has been shown to up-regulated in DENV2 infected endothelial cells [55]. *Phyllanthus* may have been suppressing these proteins to inhibit viral replication; however this remains to be addressed.

## Conclusions

From our in-vitro experiments, we noted that *Phyllanthus* does have antiviral activities against DENV2 and this was further supported by differential regulation of various host and viral proteins (Figure 5). We also propose that *Phyllanthus* is an early inhibitor as it showed most anti-DENV effect prior to infection or during infection. Our suggestion is strengthened by the fact that the plant targeted 13 differentially regulated proteins which were of the cell-virus attachment, viral entry, viral polyprotein production, viral RNA replication as well as viral assembly and maturation. We envisage that the aqueous extract of *Phyllanthus* has the potential to be a candidate in the development of anti-dengue agents, however, additional work involving the other DENV serotypes, *in vivo* (toxicity and efficacy) and pre-clinical studies need to conducted for this extract to be concluded as a good anti-viral agent.

## Competing interests

The authors declare that they have no competing interests.

## Authors’ contributions

SDS conceived of the study, participated in its design and coordination as well as helped to draft and edited the manuscript. SHL, YQT, AR, and SMW carried out the experimental works, performed the statistical analysis and drafted the manuscript. KCO, RM, and BJP performed data analysis and edited the manuscript. IBJ carried out extracts preparation and edited the manuscript. All authors read and approved the final manuscript.

## Pre-publication history

The pre-publication history for this paper can be accessed here:

http://www.biomedcentral.com/1472-6882/13/192/prepub

## Supplementary Material

Additional file 1 **Description of Data: The in vitro viral activity of aqueous *Phyllanthus *cocktail at 3 different treatment modes (pre-, simultaneous-, post-) were analyzed using real time qPCR technique, whereby simultaneous mode of treatment showed strongest antiviral activity against DEN-2 virus.** Click here for file
